# Biomarker-Drug and Liquid Biopsy Co-development for Disease Staging and Targeted Therapy: Cornerstones for Alzheimer’s Precision Medicine and Pharmacology

**DOI:** 10.3389/fphar.2019.00310

**Published:** 2019-03-29

**Authors:** Harald Hampel, Edward J. Goetzl, Dimitrios Kapogiannis, Simone Lista, Andrea Vergallo

**Affiliations:** ^1^AXA Research Fund & Sorbonne University Chair, Paris, France; ^2^Sorbonne University, GRC n° 21, Alzheimer Precision Medicine (APM), AP-HP, Pitié-Salpêtrière Hospital, Paris, France; ^3^Brain & Spine Institute (ICM), INSERM U 1127, CNRS UMR 7225, Boulevard de l’Hôpital, Paris, France; ^4^Department of Neurology, Institute of Memory and Alzheimer’s Disease (IM2A), Pitié-Salpêtrière Hospital, AP-HP, Boulevard de l’Hôpital, Paris, France; ^5^Department of Medicine, University of California, San Francisco, San Francisco, CA, United States; ^6^Laboratory of Neurosciences, Intramural Research Program, National Institute on Aging, Baltimore, MD, United States

**Keywords:** Alzheimer’s disease, liquid biopsy, exosomes, systems pharmacology, precision medicine

## Abstract

Systems biology studies have demonstrated that different (epi)genetic and pathophysiological alterations may be mapped onto a single tumor’s clinical phenotype thereby revealing commonalities shared by cancers with divergent phenotypes. The success of this approach in cancer based on analyses of traditional and emerging body fluid-based biomarkers has given rise to the concept of liquid biopsy enabling a non-invasive and widely accessible precision medicine approach and a significant paradigm shift in the management of cancer. Serial liquid biopsies offer clues about the evolution of cancer in individual patients across disease stages enabling the application of individualized genetically and biologically guided therapies. Moreover, liquid biopsy is contributing to the transformation of drug research and development strategies as well as supporting clinical practice allowing identification of subsets of patients who may enter pathway-based targeted therapies not dictated by clinical phenotypes alone. A similar liquid biopsy concept is emerging for Alzheimer’s disease, in which blood-based biomarkers adaptable to each patient and stage of disease, may be used for positive and negative patient selection to facilitate establishment of high-value drug targets and counter-measures for drug resistance. Going beyond the “one marker, one drug” model, integrated applications of genomics, transcriptomics, proteomics, receptor expression and receptor cell biology and conformational status assessments during biomarker-drug co-development may lead to a new successful era for Alzheimer’s disease therapeutics. We argue that the time is now for implementing a liquid biopsy-guided strategy for the development of drugs that precisely target Alzheimer’s disease pathophysiology in individual patients.

## Challenges in Biomarker-Based Algorithm for Drug R&D Programs

The dramatic rate of Alzheimer’s disease (AD) clinical trial failures for putative disease-modifying therapies (DmT) calls for a shift in Research and Development (R&D) strategies. The drug development field needs to advance to a more comprehensive scrutinization of AD-related pathophysiology, an earlier detection and definition of appropriate preclinical target subpopulations, taking interindividual biological variability into account (Cummings, 2011; Hampel et al., 2018a).

Particularly during early disease stages, evolving decompensating molecular pathways may be effectively regulated (Hampel et al., 2018a,b). At specific time points during the long preclinical stage, brain network homeostasis as well as adaptive responses and compensatory mechanisms may still be restorable, thus ensuring resilience and ultimately prolonging brain health-span (Hampel et al., 2018a,b).

An agnostic hypothesis-independent biomarker-driven classification system (the A/T/N system) has been proposed to stratify individuals according to core AD-related pathological and pathophysiological hallmarks (brain overaccumulation of both amyloid-β and tau proteins aggregates, and neurodegeneration) (Jack et al., 2016). The A/T/N system is aligned with the established conceptual framework and is based on much-researched and largely-validated CSF and PET biomarkers of AD. The A/T/N system is expected to provide consistent subject enrollment and target engagement among different sites in AD clinical trials.

Although the ATN system provides key pathophysiological insights, it only partially reflects the expanding *spectrum* of pathomechanistic alterations occurring in AD (Hampel et al., 2018c; Molinuevo et al., 2018). Newly identified disease mechanisms may represent relevant therapeutic targets and an increasing number of such innovative compounds are currently investigated by industry-led R&D programs (Hampel et al., 2018a; Molinuevo et al., 2018).

A growing number of candidate biomarkers, capable of charting a wider set of age- and AD-related pathophysiological pathways is currently available, for instance: upstream regulators of amyloidogenic pathways and axonal sprouting such as β-site amyloid precursor protein (APP) cleaving enzyme 1 (BACE1) (Shen et al., 2018), TAR DNA binding protein-43 (TDP-43) (Molinuevo et al., 2018), regulators of astrocyte- and microglia-mediated dysfunction and neuroinflammation (such as the human cartilage glycoprotein-39 and chitinase-like protein 1, [YKL-40] and TREM2) (Hampel et al., 2018c; Molinuevo et al., 2018), damage of large-caliber myelinated axons [reflected by release of neurofilament light chain (NFL)] (Mattsson et al., 2017; Preische et al., 2019), synaptic dysfunction (reflected by release of synaptic proteins, such as neurogranin and alpha-synuclein) (Kvartsberg et al., 2015; Vergallo et al., 2018).

Besides biological and pathophysiological issues, a number of challenges remain to reach the objective of translating these criteria into an operational biomarker-based algorithm for drug R&D programs as well as for broad clinical diagnostic practice.

First, there is a need to better understand the role that established, and newly identified biomarkers may play for distinctive contexts-of-use (COU), such as for clinical diagnosis, preclinical risk assessment and participant selection (O’Bryant et al., 2017).

With the global epidemic rise of AD and with the exponential increase in the number of individuals at risk and or individuals with preclinical disease, it is urgent to step up the development of comprehensive blood-based biomarker panels which are widely accessible, minimally invasive, and less time- and cost-consuming compared with CSF and neuroimaging assessment.

The collection, processing, and storage of blood is globally accessible from biotech and pharmaceutical industry, to academic research, until primary health care facilities. All advantages of blood-based biomarkers compared with CSF or PET biomarkers are essential in clinical research studies investigating large-scale heterogeneous populations of cognitive normal individuals at risk of AD and when time series are part of the study design.

The roadmap to ultimately validating and qualifying blood-based biomarkers for different COU and to set up a liquid biopsy for AD still requires an enormous worldwide-scaled effort for the standardization and harmonization of preanalytical and analytical protocols.

In this regard, a professional interest area focused on Blood Based Biomarkers (BBB-PIA), as a part of the Alzheimer’s Association’s International Society to Advance Alzheimer’s Research and Treatment (ISTAART) as well as the European Union-North American Clinical Trials in Alzheimer’s Disease Task Force (EU/US CTAD Task Force) are intensely supporting the achievement of a field-wide consensus on the harmonization of pre-analytic and analytic protocols in order to speed up the analytical and clinical validation of blood-based biomarkers (Snyder et al., 2014).

In the last 5 years, several population-based studies demonstrated correlation between CSF and blood concentrations of different candidate markers. Moreover, several mono- and multi-centric studies have proven a significant predictive power of blood-based biomarkers of brain amyloidosis and tau-related neuronal injury using PET and/or MRI imaging as a standard of truth (Fandos et al., 2017; Mielke et al., 2018; Nakamura et al., 2018; Park et al., 2019).

Therefore, a blood-based matrix would help overcome the invasive and time-consuming profile of lumbar puncture and would display an intrinsic superiority compared with PET-based methods that can capture only a single molecular mechanism at a time.

Such a comprehensive blood-based biomarker matrix has already become reality in more advanced biomedical areas including Oncology and clinical Immunology constituting the liquid biopsy approach (Wan et al., 2017; Heitzer et al., 2019; Pantel and Alix-Panabieres, 2019).

The U.S. National Cancer Institute (NCI) defines liquid biopsy (LB) as “a test done on a sample of blood to look for cancer cells from a tumor that are circulating in the blood or for pieces of DNA from tumor cells that are in the blood”.

The progressive field of Oncology is increasingly benefiting from breakthrough high-resolution biomarker technologies generating liquid biopsy-driven patient health care algorithms and successfully implementing anti-cancer drug R&D pipelines.

Anti-cancer clinical trials are increasingly employing the exhaustive biological tumor profiling facilitated by liquid biopsy, which paves the way toward a paradigmatic shift to precision medicine and pathway-based medicine. In 2017 the U.S. Food and Drug Administration (FDA) had accelerated the approval of two tumor type- and site- agnostic therapy approaches, pembrolizumab and nivolumab (Goldberg et al., 2018).

We argue that the right time to develop liquid biopsy-guided strategies for AD drug R&D pipelines is clearly approaching.

In our model we suggest that the term liquid biopsy would equally account for any trackable bioindicators originating in brain tissue inflicted by the pathophysiological processes of AD. Liquid biopsy of AD will enable an early biological stratification of large-scale heterogeneous populations of asymptomatic individuals at risk and patients with preclinical AD. In a future multi-stage risk-population screening strategy, liquid biopsies may identify individuals at higher risk for AD who may be further investigated with more specific, cost-intensive, and invasive methods (such as CSF or PET investigations) (Hampel et al., 2018c; Molinuevo et al., 2018).

Several blood-based candidate biomarkers reflecting distinct pathophysiological mechanisms of AD as well as unbiased exploratory quantitative high-throughput “omics” biomarker platforms are increasingly gaining *momentum*. We hypothesize that Neural-Derived Exosome Protein and MicroRNAs (miRNAs) may constitute the scaffolding of a single standardized workflow to (I) expand the current research criteria of AD with additional molecular biomarkers belonging to the wider spectrum of the pathophysiological landscape of polygenic late-onset AD, (II) expand the unbiased A/T/N biomarker classification system to integrate comprehensive pathophysiological blood-based biomarker information, making real-world application of this application more feasible, cost- and time-effective and globally accessible.

## Neural-Derived Exosome Protein Abnormalities Reveal Targets for Prevention and Therapy of CNS Diseases in the Elderly: Neural Exosome Protein Therapeutic Targets

An emerging possibility for prediction of preclinical Alzheimer disease (AD), other neurodegenerative diseases and cerebrovascular disease is quantification of pathogenic proteins in plasma neural cell-derived exosomes (Mustapic et al., 2017). We will review recent progress in neural exosome analysis, advances in clinical applications, and examples of exosome protein biomarker candidates that may become targets for preventative or therapeutic approaches to CNS diseases.

Exosomes derived from any type of CNS cell may be harvested efficiently from human plasma by initial physical precipitation followed by immunoabsorption with antibodies that selectively recognize a surface antigen on that CNS cell-type (Mustapic et al., 2017). This two-step process permits separate enrichment of exosomes secreted by neurons, astrocytes, microglia and chondroitin sulfate proteoglycan type 4 (CSPG4) cells.

The evidence for CNS cell-origin of exosomes recovered through this process continues to accumulate. Briefly, co-authors in this paper and others have demonstrated many-fold higher levels for multiple known neuronal and astrocytic markers using Western Blot and ELISA-type analyses (Goetzl et al., 2016; Mustapic et al., 2017); enrichment for multiple neuronal proteins based on targeted and untargeted proteomics (Pulliam et al., 2019) and demonstration of high-yield immunocapture of known neuronal exosomes from the plasma matrix [see Supplementary Material in Athauda et al. (2019)]. Efforts to directly determine the relationship between brain pathology and circulating exosomes in animal models and humans are currently underway.

In steady-states of chronic CNS degeneration, the plasma concentration of exosomes from each cell-type are unchanged by disease and neuropathological effects are seen predominantly in the concentrations of cargo proteins per exosome (Mustapic et al., 2017). Variations in efficiency of neural exosome isolation from individual plasma are determined using constitutive protein markers present at uniform levels irrespective of disease. Normalization of biomarker levels to the same number of exosomes with constitutive markers [such as CD81 (Fiandaca et al., 2015) or Alix (Pulliam et al., 2019)] delineates effects of CNS diseases. Progress using this approach includes the following findings:

(1) Higher levels of P-T181-tau, P-S396-tau and Aβ42 in neuronally-derived exosomes (NDEs) of patients with AD compared to age-matched controls, that were detected as early as 10 years before overt clinical disease (Fiandaca et al., 2015).
(2) NDE protein abnormalities characteristic of defective autophagy, insulin resistance, synaptic dysfunction, and reduced protection against cellular stress in AD (Goetzl et al., 2018a).
(3) Substantially lower intracellular levels of the Aβ peptide-generating proteins, such as β-secretase cleaving enzyme 1 (BACE-1) γ-secretase and APP in astrocytes compared to neurons, but much higher secreted concentrations of these proteins in astrocyte-derived exosomes (ADEs) compared to NDEs, with ADEs of AD patients having higher levels compared to controls (Goetzl et al., 2016).
(4) Higher concentrations of neurotoxic complement proteins, such as C3b, Bb and C5b-C9, and lower levels of endogenous complement inhibitors, such as CD59, in ADEs of AD patients compared to controls (Goetzl et al., 2018b).
(5) Higher NDE concentrations of P-T181-tau, P-S396-tau and Aβ42, and ADE levels of complement proteins in subjects with mild cognitive impairment (MCI) predictive of conversion to AD dementia in three years with high sensitivity.
(6) Higher endothelial cell-derived exosome (EDE) concentrations of CNS-specific endothelial proteins in patients with clinical cerebrovascular disease compared to controls (Goetzl et al., 2017).
(7) Higher EDE levels of CNS-specific endothelial proteins and cytotoxic complement proteins in asymptomatic subjects with MRI signs of small vessels cerebrovascular disease compared to age-matched controls having normal MRIs.

Tentative evaluation of our neural-derived exosome biomarker data suggests potential value of some cargo proteins as therapeutic targets (Table 1). In AD, the highest value may be assigned to blocking astrocyte complement-mediated neurotoxicity and to restoring growth and regenerative factors of the small, but widespread, set of CSPG4 cells. Altering activities of various kinases and proteases and restoring levels of excitatory synaptic proteins may also be useful efforts, but lack of specificity is a deterrent. In cerebrovascular disease, similar efforts to block complement-mediated endothelial toxicity are promising. Enhancing eNOS activity in endothelial cells by suppressing the level of NOSTRIN and enhancing angiogenesis of collateral vessels by increasing endothelial P-YAP-1 transcriptional activity provide optimism.

**Table 1:** Experimental therapeutic targets suggested by neural exosome protein abnormalities.

Disease	Protein(s)	Neural cell source	Desired effect	Estimated feasibility as an approachable target
AD	P-tau species	Neurons	Suppress kinase(s) (CDK5, GSK3b, ERK2)	1
			Enhance phosphatase(s)	
	P-S-IRS-1	Neurons	Suppress kinase(s)	2
	Excitatory pathway synaptic proteins	Neurons	Restore protein levels	2
	Aβ-generating BACE-1 and γ-secretase	Astrocytes	Selective protease inhibitors	2
	Complement (C) system [C3b, Bb, C5b-C9]	Astrocytes	Suppress complement activation; block C receptors	3
	Growth and regenerative factors (HGF, FGF-13, IGF-1)	CSPG4 cells	Restore CSPG4 proteins	3
CeVD	Regulators of vascular tone and repair/regeneration (NOSTRIN, YAP-1/P-YAP-1)	Endothelial cells	Decrease NOSTRIN, increase active YAP-1	2
	Complement (C) system [C3b, Bb, C5b-C9]	Endothelial cells	Suppress complement activation; block C receptors	3


*AD, Alzheimer disease; CeVD, cerebrovascular disease; P-S-IRS-1, serine phosphorylated type 1 insulin receptor substrate; Aβ, amyloid β peptide; BACE-1, type 1 β-secretase or type 1 β site-amyloid precursor protein cleaving enzyme; CSPG4 cell, neural cell type expressing high levels of chondroitin sulfate proteoglycan; HGF, hepatic growth factor; FGF-13, type 13 fibroblast growth factor; IGF-1, type 1 insulin growth factor; NOSTRIN, nitric oxide synthase traffic inducer; YAP-1, type 1 Yes-associated protein; P-YAP, S127-phosphorylated YAP1. The estimated feasibility scale is: 1 = unlikely to succeed, 2 = likely to succeed if technical obstacles are overcome, 3 = likely to be a useful approach.*

## MicroRNA–Messenger RNA (miRNA–mRNA) Signalling in Alzheimer’s Disease

MicroRNAs (miRNAs) constitute a large family of small non-coding RNA, consisting of maximum 24 nucleotides, that exert an inhibitory function of gene expression, by destabilizing messenger RNAs and down-regulating the translation process (Alexandrov et al., 2012; Zhao et al., 2016). Consequently, miRNAs may play a key regulatory role for all intracellular signals.

The deregulation of miRNAs turn-over is associated with the development of a broad *spectrum* of diseases such as cancer, diabetes, and several pathological brain conditions including multiple sclerosis and sporadic AD (Alexandrov et al., 2012; Bergman et al., 2016; Sorensen et al., 2016; Zhao et al., 2016).

Research work to date has defined a small ∼5-membered family of NF-kB-inducible miRNAs including miRNA-9, miRNA-34a, miRNA-125b, miRNA-146a and miRNA-155 which have been found to be up-regulated in the extracellular and cerebrospinal fluid contiguous to AD-affected brain regions (Alexandrov et al., 2012; Bergman et al., 2016; Sorensen et al., 2016; Zhao et al., 2016). These results have been found in independent studies conducted on autopsy-confirmed AD patients, transgenic murine models of AD (TgAD), and stressed human brain cells derived from primary cultures. These up-regulated miRNAs appear to slow down their mRNA targets, providing a mechanistic explanation for several interactive AD-related failures involving phagocytosis, synaptogenesis, bioenergetic homeostasis, modulation of inflammatory signaling, and amyloidogenesis (Alexandrov et al., 2012; Bergman et al., 2016; Sorensen et al., 2016; Zhao et al., 2016). For example, the up-regulation of both miRNA-9 and miRNA-146a in AD has been shown to down-regulate the expression of the innate-immune glycoprotein complement factor H (CFH), and this stimulates runaway innate-immune and inflammatory signalling in AD that directly impacts mitochondrial-based energy-generating capacities (Alexandrov et al., 2012; Bergman et al., 2016; Sorensen et al., 2016; Zhao et al., 2016).

Whether these circulating miRNAs predict highly specific genetic-epigenetic signalling pathways involved in the pathophysiology of sporadic AD as well as already established fluid and neuroimaging biomarkers, will be clarified in the coming years and may open up a therapeutic avenue to molecular miRNA-targeted therapies.

## Liquid Biopsy-Guided Drug Development

Systems biology derived exploratory biomarkers are beginning to bridge the historically developed separation between medical specialties and the deconstructed disease concepts as well as traditionally delineated drug development programs (e.g., within and between the different fields) (Hampel et al., 2018a,b). Indeed, biomarker panels are untangling shared pathways (pathophysiological and pathological commonalities) between nosologically diverse diseases which are differently conceptualized across different medical fields, such as Neurology, Oncology, and clinical Immunology.

**FIGURE 1:**
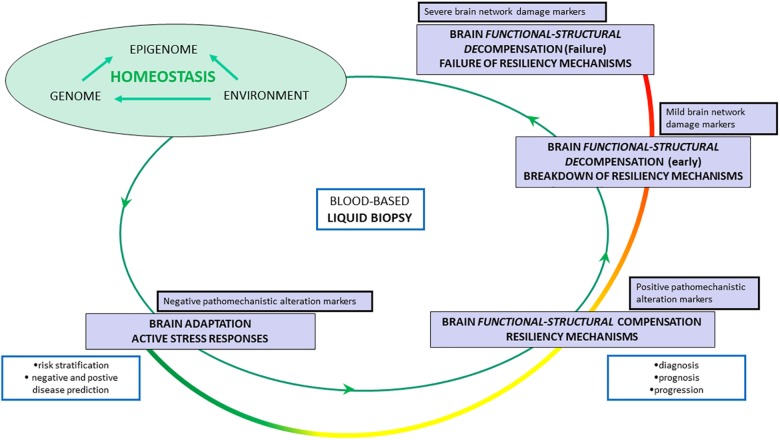
Liquid biopsy-guided management of Alzheimer’s disease according to a hypothetical model of spatial-temporal system-wide shifts: from adaptation to irreversible failure. Homeostasis is ensured by adaptive responses and compensatory mechanisms scaled in time and space across multi-level system networks: from molecular pathways, to cellular stress responses, to brain cell-to-cell and synaptic dynamics, to large-scale brain network activity, to brain-periphery cross-talks. If a decompensatory cascade occurs, homeostasis progressively breaks down until final systems failure. *Functional stage – adaptation stage – stress responses*: Metabolic and energetic reconfiguration associated with functional switch in molecular/cellular/tissue/brain systems/body system network activity. *Functional–structural stage – compensation stage – resiliency mechanisms*: Structural and functional counterbalancing of one or more initial pathomechanistic alterations. *Early decompensation stage – breakdown of resiliency mechanisms*: Initial and progressive loss of compensatory effect (resilience) or over sustained compensation which may be neither protective nor homeostatic. *Late decompensation stage (failure stage) – failure of resiliency mechanisms*: Hypothetical point of no-return. The colored curve line from green to red indicates a decreasing magnitude of drugs efficacy. The colored curve from green to red indicates a decreasing magnitude of drugs efficacy. The blue rectangles indicate the different context-of-use of biomarkers according to the pathophysiological evolution.

Liquid biopsies will define stage- and individual-specific thresholds for AD pathophysiological dynamics and will constitute the core of future disease modeling paradigms in which different biological levels can be explored simultaneously (Roukos, 2017; Hampel et al., 2018c). Indeed, we hypothesize that liquid biopsy will enable to untangle the spatial and temporal dynamics between molecular-cellular pathways and brain large-scale brain network functional shifts, from adaptation to resilience until systems failure and cognitive decline. Hypothesis-independent identification and quantification of evolving risk of detrimental mechanisms of AD will be facilitated through a multi-purpose biomarker *armamentarium* (see Figure 1).

A liquid biopsy-guided stratification approach based on upcoming clustering and other artificial intelligence methods will create novel patient strata aggregating subjects which have similar biomarker profiles and segregating patients which do not.

To follow, the clinical trajectories of each biological cluster will be mapped out, hence, providing an innovative liquid biopsy-based risk prediction matrix of AD pathophysiology in asymptomatic at-risk individuals. Moreover, liquid biopsies may inform a staging framework from very early pathophysiological alterations to later preclinical and clinical manifestations. To achieve these objectives, it is necessary to trace and model individual longitudinal trajectories and dynamics for each biomarker taking into account age ranges, sex, and common genetic variants associated with increased risk of developing AD (Nho et al., 2013; Ferretti et al., 2018; Pimenova et al., 2018).

The catalyzing impact of systems theory, systems biology, systems pharmacology, and precision pharmacology-oriented drug R&D programs will ultimately support more precise future medical strategies (Hampel et al., 2018a,b).

Systems pharmacology is a novel conceptual framework that models traditional pharmacological parameters, derived from pharmacodynamics and pharmacokinetics, operating under the theoretical background of systems medicine (Geerts et al., 2015, 2018). Systems pharmacology allows modeling a drug effects through key biological factors such as the genetic background, sexual dimorphism and age-related pathomechanistic alterations (Geerts et al., 2015; Roberts et al., 2016). Precision pharmacology is a biomarker-based approach providing pathway-based therapies through exploratory and predictive outcomes, from initial proof-of-pharmacology to all subsequently relevant decision-making processes (Hampel et al., 2018d).

We argue that liquid biopsy will support the proof of pharmacology for a putative DmT which requires successful demonstration of the capacity of a compound on several critical levels. This means clear indication of engagement of the intended biological target (i.e., druggability: the capacity of a molecular target to be modulated by a small-molecule drug), with predictable downstream alterations in pathophysiological mechanisms, in association with clinically relevant benefits (Geerts et al., 2015; Kozakov et al., 2015; Wu et al., 2016). For instance, recent studies have provided proof of principle for using protein cargo in neural-derived exosomes to demonstrate engagement of a specific target intracellular signaling cascade (e.g., the insulin cascade) by an experimental treatment (Eitan et al., 2017; Athauda et al., 2019). In particular, signaling mediators manifesting different functionality depending on their phosphorylation state may be particularly revealing for the effects of a drug when consistent changes are revealed in neural-derived exosomes after treatment. Such a signal has been likened to a “message in a bottle” (Dubal and Pleasure, 2019).

It is well established that multifactorial polygenic diseases such as several cancers, diabetes and AD, show non-linear pathophysiological dynamics likely due to evolutionary conserved pathways with complex molecular cross-talks and feedback loops and may also manifest individual differences in drug response (Rask-Andersen et al., 2014; Mitsopoulos et al., 2015; Deng and Nakamura, 2017; Roukos, 2017). Thus, in cancer as well diabetes and brain diseases, the magnitude of the improvement in clinical outcomes attained by a given small molecule may substantially change over time depending on the stage of the disease and the individual (epi)genetic biological background. Indeed, the likelihood of a positive biological effect induced by specific drug on a specific target may change over time, being related to the spatial-temporal target druggability patterns (dynamic evolution of the ligand binding affinity) as well as to mechanisms of drug resistance (Rask-Andersen et al., 2014; Mitsopoulos et al., 2015; Deng and Nakamura, 2017; Roukos, 2017). Drug-resistance refers to the presence of gene variants that impact drug target pathways through downstream effectors with either gain or loss of function. Within biomarker-positive populations entering a trial of a molecularly targeted treatment identified as druggable, some individuals are expected to display primary or acquired resistance (Schubbert et al., 2007; Deng and Nakamura, 2017; Drilon et al., 2018).

**FIGURE 2:**
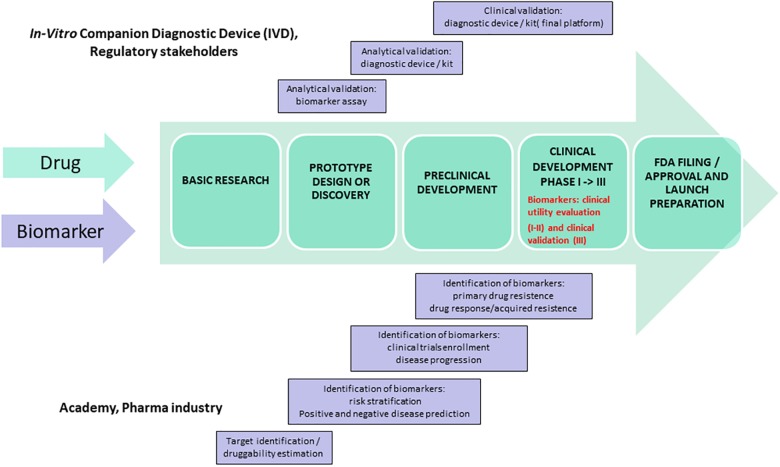
Ideally, biomarkers should be carried through all phases of drug development and validated and qualified in agreement with regulators. The picture shows one possible model for biomarker-drug co-development program: a regulatory scenario for a single test that would be used in conjunction with a single drug in the clinical management of a patient. The figure highlights key events for both the diagnostic test and drug regulation with overall coordination of the regulatory processes governing them so that the products launch together.

Temporary patterns of target druggability may be tightly linked to some key spatial and temporal pathophysiological coordinates that today can be deciphered through experimental and computational innovation integrated in the systems medicine approach (Roberts et al., 2016; Geerts et al., 2018). Likewise, cancer also in AD, liquid-biopsy will serve to accomplish druggability and drug-resistance profiling, from *in silico* computational prediction to *in vivo* biomarkers-drug co-development programs with the approval of both *in vitro* companion diagnostic device (IVD) and hopefully effective pharmacological compounds (see Figure 2) (Jorgensen and Hersom, 2016; Twomey et al., 2017). The feasibility profile of liquid-biopsy (minimally invasive, cost- and time-effective) seems to perfectly match the requirements of academic researchers, pharma companies, and regulatory stakeholders to successfully inform biomarker-drug co-development programs (Nicolaides et al., 2014; Jorgensen and Hersom, 2016; Twomey et al., 2017; Hampel et al., 2018c). This process will hopefully translate into discovering novel targets, including some currently considered non-druggable due to the lack of adequate surrogate outcomes. Consequently, liquid-biopsy related biomarker-drug co-development will improve the blueprints of clinical trials for AD by facilitating adaptive designs.

We assume that liquid biopsy may represent a key tool of systems pharmacology to dynamically and adaptively prioritize potential drug targets consistent with the pathophysiological evolution of the disease. Such focused breakthrough will support the precise treatment of the right patient, the best druggable target(s) at a defined time point of the disease based on a biologically identified and classified stage.

## Conclusion

Biomarker panels critically support objective clinical decision-making processes in health facilities, academic research settings, payor organizations, and pharma/biotech development programs (O’Bryant et al., 2017; Hampel et al., 2018a).

Current efforts to re-define diagnostic criteria and bolster programs for biomarker discovery and drug development based on liquid-biopsy-guided disease modelling show promise to lead us to precision medicine for AD (Nicolaides et al., 2014; Jorgensen and Hersom, 2016; Twomey et al., 2017; Hampel et al., 2018c).

Clinical cancer studies investigating pathway-based therapies have shown, however, that not all biomarker-positive patients respond to a given compound (Drilon et al., 2018). Both positive and negative predictive biomarkers are required for detecting and quantifying target druggability and drug resistance, guiding the selection of patient subsets for specific treatments, and for dynamically treating AD as its pathophysiology evolves (Geerts et al., 2018; Gibson, 2019). To validation, qualification, and standardization of a comprehensive liquid biopsy for AD, blood-based biomarker panels should be subjected to rigorous testing using large-scale observational and longitudinal studies of cognitively intact individuals at risk of AD.

Toward this end, consequent academic-industry blood-based biomarker- drug co-development collaboration programs through all development stages and with strong regulatory science consideration are required. The Interest Area focused on Blood Based Biomarkers (BBB-PIA) - integrated in the Alzheimer’s Association’s ISTAART, in close collaboration with the Alzheimer Precision Medicine Initiative (APMI ^1^) – will continue to facilitate the stepwise identification, validation, and standardization process of biomarker candidates (O’Bryant et al., 2017; Hampel et al., 2018c).

These advances are required to facilitate the emerging precision pharmacology and precision medicine paradigms in neuroscience and neurology, as already successfully established in other medical fields.

The emerging liquid biopsy framework is by definition not restricted to any disease field or specialty, it is an approach facilitated by technological and conceptual advances in the discovery and validation of blood-based biomarkers indicative of pathophysiological mechanisms underlying any disease. Therefore, it becomes inherently clear that the current substantial advances in blood-based biomarker development in AD will subsequently lead into practical and useful liquid biopsy solutions as part of early detection and screening as well as drug discovery programs. These solutions are currently on the horizon, based on strong evolving biomarker evidence.

## Members of the Alzheimer Precision Medicine Initiative – Working Group (APMI–WG)

Mohammad Afshar (Paris), Lisi Flores Aguilar (Montréal), Leyla Akman-Anderson (Sacramento), Joaquín Arenas (Madrid), Claudio Babiloni (Rome), Filippo Baldacci (Pisa), Richard Batrla (Rotkreuz), Norbert Benda (Bonn), Keith L. Black (Los Angeles), Arun L. W. Bokde (Dublin), Ubaldo Bonuccelli (Pisa), Karl Broich (Bonn), Francesco Cacciola (Siena), Filippo Caraci (Catania), Juan Castrillo (deceased) (Derio), Enrica Cavedo (Paris), Roberto Ceravolo (Pisa), Patrizia A. Chiesa (Paris),Jean-Christophe Corvol (Paris), Augusto Claudio Cuello (Montréal), Jeffrey L. Cummings (Las Vegas), Herman Depypere (Gent), Bruno Dubois (Paris), Andrea Duggento (Rome), Enzo Emanuele (Pavia), Valentina Escott-Price (Cardiff), Howard Federoff (Irvine), Maria Teresa Ferretti (Zurich), Massimo Fiandaca (Irvine), Richard A. Frank (Malvern), Francesco Garaci (Rome), Hugo Geerts (Berwyn), Filippo S. Giorgi (Pisa), Edward J. Goetzl (San Francisco), Manuela Graziani (Roma), Marion Haberkamp (Bonn), Marie-Odile Habert (Paris), Harald Hampel (Paris), Karl Herholz (Manchester), Dimitrios Kapogiannis (Baltimore), Eric Karran (Cambridge), Steven J. Kiddle (Cambridge), Seung H. Kim (Seoul), Yosef Koronyo (Los Angeles), Maya Koronyo-Hamaoui (Los Angeles), Todd Langevin (Minneapolis-Saint Paul), Stéphane Lehéricy (Paris), Alejandro Lucía (Madrid), Simone Lista (Paris), Jean Lorenceau (Paris), Dalila Mango (Rome), Mark Mapstone (Irvine), Christian Neri (Paris), Robert Nisticò (Rome), Sid E. O’Bryant (Fort Worth), Giovanni Palermo (Pisa), George Perry (San Antonio), Craig Ritchie (Edinburgh), Simone Rossi (Siena), Amira Saidi (Rome), Emiliano Santarnecchi (Siena), Lon S. Schneider (Los Angeles), Olaf Sporns (Bloomington), Nicola Toschi (Rome), Steven R. Verdooner (Sacramento), Andrea Vergallo (Paris), Nicolas Villain (Paris), Lindsay A. Welikovitch (Montréal), Janet Woodcock (Silver Spring), Erfan Younesi (Esch-sur-Alzette).

## Author Contributions

HH conceptualized the study, wrote the article, and provided a critical review of the literature. SL, EG, and DK contributed to the writing of the article and provided a critical review of the literature. AV conceptualized the study, contributed to the writing of the article, and provided a critical review of the literature.

## Conflict of Interest Statement

SL received lecture honoraria from Roche. HH serves as Senior Associate Editor for the Journal Alzheimer’s & Dementia; he received lecture fees from Biogen and Roche, research grants from Pfizer, Avid, and MSD Avenir (paid to the institution), travel funding from Functional Neuromodulation, Axovant, Eli Lilly and company, Takeda and Zinfandel, GE-Healthcare and Oryzon Genomics, consultancy fees from Jung Diagnostics, Qynapse, Cytox Ltd., Axovant, Anavex, Takeda and Zinfandel, GE Healthcare and Oryzon Genomics, and Functional Neuromodulation, and participated in scientific advisory boards of Functional Neuromodulation, Axovant, Eli Lilly and company, Cytox Ltd., GE Healthcare, Takeda and Zinfandel, Oryzon Genomics and Roche Diagnostics. He is co-inventor in the following patents as a scientific expert and has received no royalties:

• *In Vitro* Multiparameter Determination Method for The Diagnosis and Early Diagnosis of Neurodegenerative Disorders Patent Number: 8916388.
• *In Vitro* Procedure for Diagnosis and Early Diagnosis of Neurodegenerative Diseases Patent Number: 8298784. Neurodegenerative Markers for Psychiatric Conditions Publication Number: 20120196300.
• *In Vitro* Multiparameter Determination Method for The Diagnosis and Early Diagnosis of Neurodegenerative Disorders Publication Number: 20100062463.
• *In Vitro* Method for The Diagnosis and Early Diagnosis of Neurodegenerative Disorders Publication Number: 20100035286.
• *In Vitro* Procedure for Diagnosis and Early Diagnosis of Neurodegenerative Diseases Publication Number: 20090263822.
• *In Vitro* Method for The Diagnosis of Neurodegenerative Diseases Patent Number: 7547553. CSF Diagnostic *in vitro* Method for Diagnosis of Dementias and Neuroinflammatory Diseases Publication Number: 20080206797.
• *In Vitro* Method for The Diagnosis of Neurodegenerative Diseases Publication Number: 20080199966.
• Neurodegenerative Markers for Psychiatric Conditions Publication Number: 20080131921.

The remaining authors declare that the research was conducted in the absence of any commercial or financial relationships that could be construed as a potential conflict of interest.

## Footnotes

1 www.apmiscience.com
